# Association Between Thoracic Inlet Size and Cervical Anastomosis Outcomes in Esophageal Cancer Surgery

**DOI:** 10.3390/curroncol33060353

**Published:** 2026-06-11

**Authors:** Iskan Calli, Ibrahim Dogan, Halil Alper Bozkurt, Mehmet Kadir Bartin, Ezgi Sonmez, Sebahattin Celik

**Affiliations:** 1 Department of General Surgery, Faculty of Medicine, Van Yüzüncü Yıl University, Van 65080, Türkiye; 2 Department of General Surgery, Van Training and Research Hospital, University of Health Sciences, Van 65300, Türkiye; ibrahim.dogan8@saglik.gov.tr (I.D.); mehmetkadir.bartin@sbu.edu.tr (M.K.B.); ezgi.sonmez1@sbu.edu.tr (E.S.); sebahattin.celik@sbu.edu.tr (S.C.); 3 Department of General Surgery, Çam and Sakura Training and Research Hospital, University of Health Sciences, Istanbul 34480, Türkiye; halilalper.bozkurt@saglik.gov.tr

**Keywords:** esophageal cancer, posterior mediastinal reconstruction, thoracic inlet area, cervical anastomosis, anastomotic leakage, postoperative mortality

## Abstract

Esophageal cancer surgery is associated with high morbidity and mortality, and anastomotic leakage remains a critical determinant of outcomes. Although thoracic inlet size has been linked to complications in retrosternal reconstruction, its role in posterior mediastinal reconstruction is unclear. In this retrospective study, 67 patients who underwent esophagectomy with posterior mediastinal reconstruction were analyzed. Thoracic inlet measurements were obtained from preoperative computed tomography. Postoperative complications occurred in 29.9% of patients, anastomotic leakage (AL) in 14.9%, and overall 30-day postoperative mortality in 13.4%. The thoracic inlet area (TIA) was significantly lower in non-survivors than in survivors (513.5 vs. 703.3 mm^2^, *p* = 0.012). Anastomotic leakage was markedly more frequent among non-survivors (66.7% vs. 6.9%). In multivariable analysis adjusted for age and sex, thoracic inlet area remained associated with postoperative mortality; however, the findings should be interpreted cautiously because of the limited sample size and retrospective design. These findings suggest a possible association between thoracic inlet geometry and postoperative outcomes in posterior mediastinal reconstruction; however, larger prospective multicenter studies are required before definitive clinical conclusions can be drawn.

## Simple Summary

Esophagectomy remains one of the most complex surgical procedures in gastrointestinal oncology and is associated with considerable postoperative morbidity and mortality. Anastomotic leakage is among the most serious complications and may substantially worsen postoperative recovery, prolong hospitalization, and negatively affect survival. Previous studies have suggested that thoracic inlet anatomy may influence anastomotic outcomes, particularly in retrosternal reconstruction; however, its relevance in posterior mediastinal reconstruction remains poorly understood. Most investigations evaluating thoracic inlet dimensions have focused on retrosternal reconstruction, where the gastric conduit passes through a relatively confined anatomical space. In contrast, evidence regarding the potential impact of thoracic inlet geometry on outcomes after posterior mediastinal reconstruction is limited. In this study, we evaluated whether preoperative thoracic inlet measurements obtained from routinely performed computed tomography scans were associated with postoperative outcomes after esophagectomy with cervical anastomosis. Patients with smaller thoracic inlet areas tended to have higher postoperative mortality, while anastomotic leakage was markedly more frequent among non-survivors. Although the findings do not establish a direct causal relationship, they suggest that thoracic inlet geometry may represent one of several anatomical factors potentially influencing postoperative outcomes. Because thoracic inlet measurements can be obtained from standard preoperative imaging without additional cost or invasive procedures, they may contribute to future risk assessment strategies when combined with other clinical, anatomical, and surgical factors. Further prospective multicenter studies are required to validate these findings and clarify the clinical role of thoracic inlet assessment in esophageal reconstruction.

## 1. Introduction

Esophagectomy remains one of the most technically demanding procedures in gastrointestinal oncology and continues to be associated with substantial postoperative morbidity and mortality despite advances in perioperative care and minimally invasive techniques [1,2,3,4,5,6,7]. Among postoperative complications, anastomotic leakage (AL) is one of the most clinically significant because it is strongly associated with sepsis, prolonged hospitalization, impaired recovery, and postoperative mortality [8,9,10,11,12,13]. Although cervical anastomosis may provide technical and oncologic advantages in selected patients, cervical AL remains a major determinant of short-term postoperative outcome following esophageal reconstruction [9,10,11,12,13].

Following esophagectomy, reconstruction is commonly performed through the posterior mediastinal (PM), retrosternal (RS), or subcutaneous route. Among these approaches, the RS route has been extensively investigated with regard to thoracic inlet anatomy because the gastric conduit traverses a relatively confined osseous-soft tissue space at the thoracic inlet [14,15,16,17,18,19,20,21,22,23,24,25]. Previous studies by Mine et al., Sato et al., and Ninomiya et al. demonstrated that reduced thoracic inlet dimensions were associated with increased cervical anastomotic leakage after RS reconstruction [16,17,20]. These findings have led to the hypothesis that thoracic inlet geometry may influence conduit angulation, venous drainage, microperfusion, and anastomotic healing. In addition, intraoperative perfusion-oriented studies using indocyanine green fluorescence imaging have further emphasized the importance of conduit blood flow and regional anatomical constraints during esophageal reconstruction [20,26].

However, despite the growing literature regarding thoracic inlet anatomy in RS reconstruction, data concerning the PM route remain extremely limited. Unlike the RS reconstruction, the PM route follows a more physiological pathway and may therefore be less susceptible to direct conduit compression at the thoracic inlet.

Although the posterior mediastinal route is generally considered more physiological than retrosternal reconstruction, cervical anastomosis in this setting may still be influenced by local anatomical constraints at the thoracic inlet. In particular, conduit angulation, regional tissue compression, venous drainage impairment, postoperative edema, and tension at the cervical anastomotic level may collectively affect conduit perfusion and anastomotic healing. Despite these potential anatomical and physiological interactions, the relationship between thoracic inlet geometry and postoperative outcomes in posterior mediastinal reconstruction remains insufficiently characterized.

Therefore, the aim of the present study was to evaluate whether preoperative thoracic inlet measurements obtained from computed tomography are associated with postoperative outcomes in patients undergoing cervical anastomosis through the posterior mediastinal route. Specifically, we investigated the relationship between thoracic inlet geometry and postoperative complications, anastomotic leakage, and 30-day postoperative mortality following esophagectomy.

Importantly, the present study was not designed to determine the exact postoperative position of the anastomosis relative to the thoracic inlet. Rather, thoracic inlet geometry was evaluated as a potential anatomical factor that may influence conduit transposition through the cervicothoracic junction, conduit angulation, venous drainage, edema tolerance, and cervical reconstruction mechanics.

## 2. Materials and Methods

Following approval by the Ethics Committee of Van Yüzüncü Yıl University (approval code: 2024/13-17; approval date: 20 December 2024), a retrospective multicenter analysis was conducted on patients who underwent esophageal cancer surgery between January 2021 and March 2025 at two tertiary referral centers. The study was performed in accordance with the principles of the Declaration of Helsinki.

Adult patients (>18 years) who underwent esophagectomy with posterior mediastinal reconstruction and cervical anastomosis were eligible for inclusion. Demographic, perioperative, operative, and postoperative variables were retrospectively collected from institutional databases and electronic medical records. Collected variables included age, sex, neoadjuvant therapy status, tumor location, operative approach, anastomotic technique, intraoperative transfusion requirement, postoperative complications, anastomotic leakage, hospital stay duration, and 30-day postoperative mortality.

Operative approaches included transthoracic (thoracoscopic or thoracotomy-assisted) and transhiatal esophagectomy procedures. All patients underwent posterior mediastinal reconstruction with cervical anastomosis. Tumors included in the present study were located in the upper and middle thoracic esophagus. Because all included patients underwent posterior mediastinal reconstruction with cervical anastomosis, patients with distal esophageal tumors requiring planned intrathoracic anastomosis, including standard Ivor Lewis-type reconstruction, were not included in the study cohort. Cervical anastomoses were performed using either hand-sewn or circular stapled techniques according to surgeon preference and intraoperative findings. When a stapled anastomosis was used, a 25 mm circular stapling device was utilized.

Postoperative mortality was defined as death occurring within 30 days after surgery. Anastomotic leakage was considered a postoperative complication; however, because of its major clinical importance, it was additionally analyzed as a separate outcome variable. Management of anastomotic leakage was individualized according to clinical severity and included conservative treatment, drainage procedures, endoscopic interventions, or surgical revision when clinically indicated.

### 2.1. Radiological Measurements

**Figure 1:**
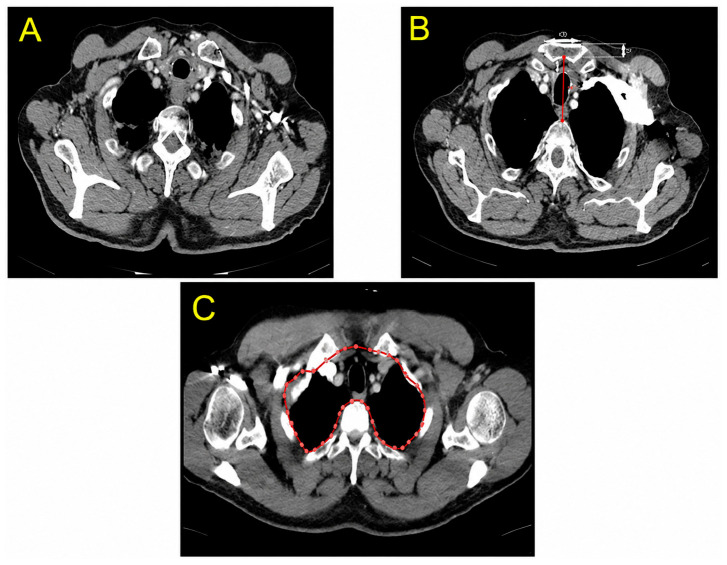
Radiologic measurement of thoracic inlet parameters on preoperative computed tomography images. (**A**,**B**) Linear measurements include sterno-tracheal distance (STD), sterno-vertebral distance (SVD), and interclavicular distance (ICD). (**C**) Thoracic inlet area (TIA) calculation based on predefined linear measurements. All images were obtained from the authors’ institutional database.

Preoperative computed tomography (CT) images were reviewed for all included patients. Measurements were obtained from axial CT images with a slice thickness of 1 mm. Radiologic parameters included interclavicular distance (ICD), sterno-tracheal distance (STD), sterno-vertebral distance (SVD), the ratio of STD to SVD (STD/SVD), and thoracic inlet area (TIA; calculated as STD × ICD) (Figure 1).

The anatomical boundaries of the thoracic inlet were defined anteriorly by the superior border of the manubrium, posteriorly by the first thoracic vertebral body (T1), and laterally by the medial borders of the first ribs. All measurements were performed by a single experienced radiologist who was blinded to postoperative outcomes.

All radiologic measurements were performed on preoperative contrast-enhanced thoracic computed tomography images obtained as part of routine preoperative staging. Measurements were reviewed using standardized anatomical landmarks to improve reproducibility and reduce observer-dependent variability. Thoracic inlet parameters were evaluated using previously described radiologic methods reported in thoracic inlet and retrosternal reconstruction studies.

Although the thoracic inlet is anatomically a three-dimensional structure, reproducible two-dimensional radiologic measurements were intentionally utilized to maintain methodological consistency with previously published thoracic inlet studies evaluating retrosternal esophageal reconstruction.

### 2.2. Statistical Analysis

Statistical analyses were performed using SPSS version 25.0 (IBM Corp., Armonk, NY, USA). Continuous variables were evaluated for normality using histograms, Q–Q plots, and formal normality testing. Because most variables were not normally distributed, continuous data were expressed as median and interquartile range (IQR), and comparisons between groups were performed using the Mann–Whitney U test. Categorical variables were expressed as numbers and percentages and were compared using the Chi-square test or Fisher’s exact test, as appropriate.

Univariate analyses were initially performed to compare survivors and non-survivors. Because anastomotic leakage was considered clinically important, its distribution according to mortality status was analyzed separately.

To evaluate whether the thoracic inlet area was independently associated with postoperative mortality, a multivariable logistic regression model was constructed, including thoracic inlet area (TIA), age, and sex. Odds ratios (ORs) with 95% confidence intervals (CIs) were calculated. Given the limited number of mortality events, the multivariable model was intentionally restricted to a limited number of covariates to reduce overfitting. All multivariable findings were therefore interpreted cautiously and considered exploratory rather than definitive. A two-sided *p*-value < 0.05 was considered statistically significant.

## 3. Results

Between January 2021 and March 2025, patients who underwent esophagectomy at the participating centers were retrospectively screened for eligibility. After exclusion of patients with incomplete radiologic imaging, missing clinical data, or failure to meet the predefined inclusion criteria, a total of 67 patients were included in the final analysis.

The median age of the study population was 55.0 years (IQR 48.5–61.5), and 42 patients (62.7%) were female. Baseline demographic, operative, and clinicopathological characteristics of the study population are summarized in Table 1. Operative approaches included transthoracic (thoracoscopic or thoracotomy-assisted) and transhiatal esophagectomy procedures. Cervical anastomoses were performed using either hand-sewn or circular stapled techniques according to surgeon preference and intraoperative findings.

The median interclavicular distance (ICD) was 28.8 mm (IQR 25.4–34.2), the median sterno-vertebral distance (SVD) was 55.2 mm (IQR 49.4–62.0), and the median thoracic inlet area (TIA) was 657.9 mm^2^ (IQR 540.3–888.2). Detailed radiologic measurements are presented in Table 2.

Postoperative complications occurred in 20 patients (29.9%). Anastomotic leakage was observed in 10 patients (14.9%), while overall 30-day postoperative mortality was 13.4% (9/67).

When patients were stratified according to mortality status, no significant differences were observed in age, ICD, or SVD. In contrast, both STD and TIA values were significantly lower in non-survivors compared with survivors (STD: 16.3 vs. 24.6 mm, *p* = 0.004; TIA: 513.5 vs. 703.3 mm^2^, *p* = 0.012). Detailed univariable comparisons according to mortality status are presented in Table 3.

Anastomotic leakage showed a strong relationship with mortality. Among the 9 patients who died, 6 (66.7%) had experienced anastomotic leakage, whereas only 4 of 58 survivors (6.9%) had leakage (*p* < 0.001). This marked difference suggests that leakage represented a major adverse postoperative event in the mortality group.

To further explore the potential relationship between thoracic inlet geometry and anastomotic leakage, thoracic inlet measurements were compared according to leakage status (Table 4). Patients who developed anastomotic leakage demonstrated lower median thoracic inlet area (TIA) values compared with those without leakage (526.8 mm^2^ vs. 682.9 mm^2^); however, the difference did not reach statistical significance (*p* = 0.111). Sterno-tracheal distance (STD) was significantly lower in patients with leakage (20.6 mm vs. 24.6 mm, *p* = 0.049), whereas no significant differences were observed for ICD, SVD, or TIA.

In multivariable logistic regression analysis including TIA, age, and sex, thoracic inlet area demonstrated an inverse association with postoperative mortality (OR 0.996, 95% CI 0.992–1.000, *p* = 0.050). Age and sex were not significantly associated with postoperative mortality in the adjusted model. Detailed multivariable regression results are presented in Table 5.

**Figure 2:**
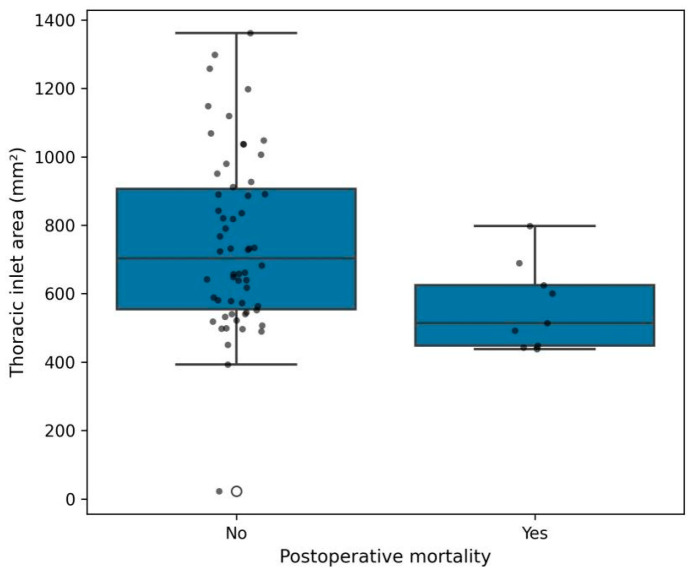
Distribution of thoracic inlet area according to 30-day postoperative mortality status. Patients with postoperative mortality demonstrated significantly lower TIA values than survivors (median 513.5 mm^2^ vs. 703.3 mm^2^, *p* = 0.012). Dots represent individual patients measurements.

The distribution of TIA according to postoperative mortality status is illustrated in Figure 2, which demonstrates visibly lower TIA values among non-survivors compared with survivors.

## 4. Discussion

The present study evaluated the relationship between thoracic inlet geometry and postoperative outcomes in patients undergoing cervical anastomosis through the posterior mediastinal reconstruction route after esophagectomy. Although thoracic inlet anatomy has previously been investigated primarily in retrosternal reconstruction, data regarding its potential relevance in posterior mediastinal reconstruction remain extremely limited. In the current cohort, smaller thoracic inlet area values were associated with postoperative mortality in univariable analyses, whereas anastomotic leakage demonstrated a particularly strong relationship with mortality. The thoracic inlet area remained associated with postoperative mortality after adjustment for age and sex; however, this finding should be interpreted cautiously because of the limited sample size and small number of mortality events.

Anastomotic leakage remains one of the most clinically important complications after esophagectomy and continues to represent a major determinant of short-term postoperative mortality despite advances in perioperative management and minimally invasive surgical techniques. Recent contemporary studies have continued to demonstrate the strong association between anastomotic leakage, septic complications, prolonged hospitalization, and postoperative mortality following esophagectomy [27,28,29,30]. In addition to its direct septic consequences, anastomotic leakage may also delay postoperative recovery, impair nutritional rehabilitation, prolong intensive care requirements, and negatively influence the timely initiation of adjuvant oncologic treatment. Therefore, prevention of leakage remains a central objective in modern esophageal surgery.

In the present study, leakage was markedly more frequent among non-survivors, further supporting the concept that anastomotic integrity remains central to postoperative outcome following esophageal reconstruction. Successful cervical anastomotic healing after esophagectomy depends on a complex interaction between conduit perfusion, tissue oxygenation, conduit tension, venous drainage, local inflammation, technical factors, and systemic physiological reserve. Even in technically successful procedures, impaired microvascular perfusion or venous congestion within the gastric conduit may contribute to ischemia at the anastomotic site and increase susceptibility to leakage. For this reason, contemporary esophageal surgery increasingly emphasizes conduit perfusion assessment and individualized reconstruction strategies aimed at reducing anastomotic complications.

Previous investigations evaluating thoracic inlet anatomy have largely focused on retrosternal reconstruction. In this setting, Mine et al., Sato et al., and Ninomiya et al. reported that reduced thoracic inlet dimensions were associated with cervical anastomotic leakage and impaired conduit perfusion parameters after retrosternal reconstruction [16,17,20]. Because the retrosternal route requires the gastric conduit to traverse a relatively confined anterior mediastinal corridor, these studies support the concept that thoracic inlet geometry may influence conduit passage, venous drainage, and anastomotic healing.

However, the posterior mediastinal route differs anatomically from retrosternal reconstruction because the conduit follows a more physiological mediastinal pathway. For this reason, direct conduit compression at the thoracic inlet may theoretically be less pronounced in posterior mediastinal reconstruction. Nevertheless, the gastric conduit differs substantially from the native esophagus in terms of wall thickness, postoperative edema susceptibility, venous congestion tendency, rotational angulation, and anastomotic bulk, particularly during the early postoperative period. Therefore, even in the absence of absolute mechanical compression, regional thoracic inlet geometry may still influence local conduit mechanics, microcirculatory dynamics, and tissue perfusion. These potential mechanisms should be interpreted as physiologically plausible but not definitively established by the present dataset.

Although the thoracic inlet area remained associated with mortality after adjustment for age and sex, the association should be interpreted cautiously because of the limited sample size and small number of mortality events. This finding is important because postoperative outcomes following esophagectomy are inherently multifactorial and are influenced by numerous perioperative, anatomical, technical, and physiological variables. Factors such as conduit preparation, anastomotic technique, cervical dissection extent, conduit tension, perfusion status, and postoperative septic complications may all contribute to anastomotic healing and mortality risk. Accordingly, the present findings should not be interpreted as evidence that thoracic inlet geometry alone determines postoperative outcome, but rather that it may represent one additional anatomical factor within a broader multifactorial risk environment.

The additional leakage-based analysis showed that patients with anastomotic leakage had lower median TIA values, although this difference did not reach statistical significance. Therefore, the relationship between thoracic inlet geometry and mortality should not be interpreted as direct causality. Anastomotic leakage likely represents a major intermediate clinical event, and thoracic inlet geometry may only be one contributory anatomical factor within a broader multifactorial risk pathway.

The present study has several strengths. To our knowledge, this is among the first studies specifically evaluating thoracic inlet geometry in patients undergoing posterior mediastinal reconstruction after esophagectomy. In addition, all radiologic measurements were performed using standardized preoperative computed tomography imaging reviewed by an experienced radiologist blinded to postoperative outcomes. Furthermore, by focusing exclusively on posterior mediastinal reconstruction, the present study provides a route-specific assessment that may facilitate comparison with the previously published retrosternal reconstruction literature. Another important aspect of the present study is the use of routinely obtainable preoperative computed tomography measurements that may be easily incorporated into standard perioperative assessment without requiring additional invasive procedures or specialized imaging protocols.

Several limitations should be considered when interpreting the present findings. First, the retrospective design may have introduced inherent selection bias and limited control over perioperative confounding variables. Second, the sample size and number of mortality events were relatively limited, which restricted the statistical power of multivariable analyses and increased the possibility of model overfitting despite cautious covariate selection. Third, a detailed quantitative assessment of conduit perfusion, conduit diameter, conduit-to-thoracic inlet ratio, and dynamic conduit compression was not available. Similarly, postoperative leakage management strategies were individualized according to clinical presentation and were therefore not standardized for comparative analysis. Although reproducible two-dimensional measurements were used to maintain methodological consistency with previous thoracic inlet studies, the thoracic inlet is anatomically a three-dimensional structure. No standardized volumetric protocol has yet been established for thoracic inlet assessment in esophageal reconstruction. Future prospective studies incorporating three-dimensional reconstruction and volumetric analysis may provide a more precise anatomical characterization. Finally, because this study focused exclusively on posterior mediastinal reconstruction, direct comparison with retrosternal reconstruction could not be performed.

From a clinical perspective, thoracic inlet assessment may represent a simple preoperative anatomical parameter that can be obtained from routinely performed staging CT scans without additional cost or imaging requirements. Although the present findings should not be used for surgical decision-making in isolation, thoracic inlet geometry may contribute to future multifactorial risk assessment models that incorporate conduit perfusion, anastomotic technique, and patient-specific anatomical characteristics.

Future prospective multicenter studies incorporating standardized conduit perfusion assessment, volumetric thoracic inlet analysis, dynamic imaging techniques, and route-specific comparisons between posterior mediastinal and retrosternal reconstruction are needed to further clarify the clinical relevance of thoracic inlet anatomy during esophageal reconstruction. In future studies, static anatomical measurements such as TIA may be integrated with dynamic functional parameters, including quantitative fluorescence perfusion metrics, conduit diameter, anastomotic configuration, and conduit-to-thoracic inlet ratios. Such integrated models may better reflect the interaction between patient-specific anatomy, conduit physiology, and surgical technique than isolated morphologic measurements alone. Recent developments in fluorescence-guided perfusion imaging and perioperative risk stratification strategies may further improve individualized surgical planning in future esophageal cancer surgery.

## 5. Conclusions

In patients undergoing cervical anastomosis through the posterior mediastinal route, smaller thoracic inlet area values were associated with postoperative mortality in univariable analysis. The thoracic inlet area remained associated with postoperative mortality after adjustment for age and sex; however, given the limited sample size and retrospective design, these findings should be interpreted cautiously and considered exploratory and hypothesis-generating rather than definitive. The present study does not establish a direct causal relationship between thoracic inlet size, anastomotic leakage, and mortality. Nevertheless, preoperative thoracic inlet assessment may represent a potentially useful adjunctive anatomical parameter in future risk stratification models.

Larger prospective multicenter studies incorporating standardized perfusion assessment and volumetric analysis are needed to further clarify the clinical relevance of thoracic inlet anatomy in esophageal reconstruction.

## Figures and Tables

**Table 1:** Baseline clinicopathological and operative characteristics of the study population.

Variable	Value
Age, median (range), years	55 (29–85)
Male sex, *n* (%)	25 (37.3)
Neoadjuvant therapy, *n* (%)	34 (50.7)
Thoracotomy, *n* (%)	27 (40.3)
Thoracoscopy, *n* (%)	11 (16.4)
Transhiatal approach, *n* (%)	29 (43.3)
Hand-sewn anastomosis, *n* (%)	30 (44.8)
Stapled anastomosis (25 mm circular stapler), *n* (%)	37 (55.2)
Operation time, median (range), minutes	180 (120–450)
Intraoperative blood transfusion, median (range), units	0 (0–2)
Anastomotic leakage, *n* (%)	10 (14.9)
30-day mortality, *n* (%)	9 (13.4)
Length of hospital stay, median (range), days	11 (6–38)

**Table 2:** Radiologic thoracic inlet measurements of the study population.

Variable	Value
Interclavicular distance (ICD), mm	28.8 (25.4–34.2)
Sterno-vertebral distance (SVD), mm	55.2 (49.4–62.0)
Thoracic inlet area (TIA), mm^2^	657.9 (540.3–888.2)
Sterno-tracheal distance (STD), mm	22.8 (19.6–29.0)

Note: Data are presented as medians (interquartile range).

**Table 3:** Univariable comparison of variables according to 30-day postoperative mortality.

Variable	No Mortality (*n* = 58)	Mortality (*n* = 9)	*p*-Value
Age, years	55.0	56.0	0.956
ICD, mm	28.6	30.8	0.452
SVD, mm	56.4	49.7	0.655
STD, mm	24.6	16.3	0.004
TIA, mm^2^	703.3	513.5	0.012
Anastomotic leakage, *n* (%)	4 (6.9%)	6 (66.7%)	<0.001

Note: Continuous variables are expressed as median values. Categorical variables are expressed as numbers (percentages). Fisher’s exact test was used for categorical comparisons when appropriate.

**Table 4:** Comparison of thoracic inlet measurements according to anastomotic leakage status.

Variable	No Leak (*n* = 57)	Leak (*n* = 10)	*p*-Value
Interclavicular distance (ICD), mm	28.8 (25.2–33.3)	30.4 (26.4–38.0)	0.403
Sterno-tracheal distance (STD), mm	24.6 (21.1–29.7)	20.6 (16.2–23.0)	0.049
Sterno-vertebral distance (SVD), mm	56.2 (49.6–62.3)	49.5 (42.8–59.3)	0.102
Thoracic inlet area (TIA), mm^2^	682.9 (563.2–889.5)	526.8 (461.3–754.9)	0.111

Note: Data are presented as median (interquartile range). Comparisons were performed using the Mann–Whitney U test.

**Table 5:** Multivariable logistic regression analysis for 30-day postoperative mortality.

Variable	OR	95% CI	*p*-Value
Thoracic inlet area (TIA)	0.996	0.992–1.000	0.050
Age	0.991	0.922–1.065	0.805
Sex (male)	1.216	0.275–5.377	0.796

Note: Odds ratios (OR) are presented with 95% confidence intervals (CI). The model includes TIA, age, and sex.

## Data Availability

Data are available from the corresponding author upon reasonable request.
